# Quantifying In Vivo Arterial Deformation from CT and MRI: A Systematic Review of Segmentation, Motion Tracking, and Kinematic Metrics

**DOI:** 10.3390/bioengineering13010121

**Published:** 2026-01-20

**Authors:** Rodrigo Valente, Bernardo Henriques, André Mourato, José Xavier, Moisés Brito, Stéphane Avril, António Tomás, José Fragata

**Affiliations:** 1 Research & Development Unit for Mechanical and Industrial Engineering (UNIDEMI), Department of Mechanical and Industrial Engineering, NOVA School of Science and Technology, NOVA University Lisbon, 2829-516 Caparica, Portugal; rb.valente@campus.fct.unl.pt (R.V.);; 2 Laboratório Associado de Sistemas Inteligentes (LASI), 4800-058 Guimarães, Portugal; 3 Inserm, Sainbiose U1059, Mines Saint-Etienne, University of Lyon, F-42023 Saint-Etienne, France; 4 Department of Cardiothoracic Surgery, Santa Marta Hospital (ULS São José), 1169-024 Lisboa, Portugal; 5 Department of Cardiothoracic Surgery, CUF Tejo, 1350-352 Lisboa, Portugal; 6 Department of Surgery and Human Morphology, NOVA Medical School, 1169-056 Lisboa, Portugal

**Keywords:** in vivo arterial deformation, biomechanical metrics, image segmentation, MRI, CT imaging, temporal registration, systematic review

## Abstract

This article presents a systematic review on methods for quantifying three-dimensional, time-resolved (3D+t) deformation and motion of human arteries from Computed Tomography (CT) and Magnetic Resonance Imaging (MRI). Following Preferred Reporting Items for Systematic Reviews and Meta-Analyses (PRISMA) guidelines, we searched Scopus, Web of Science, IEEE Xplore, Google Scholar, and PubMed on 19 December 2025 for in vivo, patient-specific CT or MRI studies reporting motion or deformation of large human arteries. We included studies that quantified arterial deformation or motion tracking and excluded non-vascular tissues, in vitro or purely computational work. Thirty-five studies were included in the qualitative synthesis; most were small, single-centre observational cohorts. Articles were analysed qualitatively, and results were synthesised narratively. Across the 35 studies, the most common segmentation approaches are active contours and threshold, while temporal motion is tracked using either voxel registration or surface methods. These kinematic data are used to compute metrics such as circumferential and longitudinal strain, distensibility, and curvature. Several studies also employ inverse methods to estimate wall stiffness. The findings consistently show that arterial strain decreases with age (on the order of 20% per decade in some cases) and in the presence of disease, that stiffness correlates with geometric remodelling, and that deformation is spatially heterogeneous. However, insufficient data prevents meaningful comparison across methods.

## 1. Introduction

Cardiovascular diseases (CVD) are the leading cause of mortality in the world, through a combination of pathologies such as stroke or aneurysms [1,2]. Those diseases are driven, mainly, by hypertension, age, and genetic predispositions that lead to the degradation of vessel walls. This leads to arterial stiffening, dilatation, and altered haemodynamics; therefore, those can be used as a definition of vascular ageing and disease [1,3]. While static information is currently used for diagnosis, it provides an incomplete picture of vascular health. The dynamic mechanical properties of arteries can offer deeper insights for clinical decision [4,5,6].

In vivo imaging is the backbone for assessing these dynamics. While Ultrasound (US) is a valuable first-line tool for dynamic metrics, its operator dependency and limited field of view limit its use for comprehensive four-dimensional (3D+t) data [7,8]. Other imaging techniques, namely CT and MRI, have emerged as alternatives for three-dimensional, time-resolved (3D+t) arterial motion. Computed Tomography Angiography (CTA) offers a significant spatial resolution for detailed geometric assessment, while MRI excels at high temporal resolution, with specialised functional analysis through techniques like cine feature tracking and 4D flow imaging [9,10,11,12,13,14]. Nonetheless, the lack of a defined methodology to extract and measure dynamic metrics limits the usability of those methods.

This paper presents a systematic review, following the PRISMA guidelines, aiming to (1) catalogue the spectrum of image acquisition, segmentation, and temporal tracking techniques; (2) synthesise the various metrics used to report deformation and stiffness; and (3) discuss the clinical implications and current limitations. In doing so, this work delivers a structured mapping and critical appraisal of existing methodologies for 3D+t arterial deformation derived from CT and MRI, highlighting converging practices, identifying methodological gaps, and proposing a prioritised set of procedures to support future standardisation efforts without asserting a definitive or exhaustive standard.

## 2. Materials and Methods

### 2.1. Search Strategy and Information Sources

A systematic literature search was conducted on 19 December 2025, across Scopus, Web of Science, PubMed, IEEE Xplore, and Google Scholar. This systematic review was conducted and reported in accordance with the PRISMA 2020 guidelines. The review protocol was registered in the Open Science Framework (OSF) (https://doi.org/10.17605/OSF.IO/PKR5G). The search covered all publications from database inception with no lower date limit applied. The following Boolean search expression was applied across all databases:

(“aorta” OR “aortic” OR “arterial” OR “vascular”) AND (“patient-specific” OR “in vivo”) AND (“strain” OR “deformation” OR “compliance” OR “motion tracking”) AND (“Computed Tomography” OR “CT” OR “Magnetic Resonance” OR “MRI”) AND NOT (“experimental” OR “Fluid-structure interaction” OR “computational fluid” OR “Finite element” OR “heart”).

Database-specific adaptations were made to accommodate syntax requirements. In Scopus, the search was conducted in the TITLE-ABS-KEY field. In Web of Science, the Topic (TS) field was used. In PubMed, MeSH terms were combined with free-text searching in Title/Abstract fields. The search was limited to peer-reviewed journal articles published in English. Conference proceedings, book chapters, editorials, and non-English publications were excluded.

This review focuses on cross-sectional imaging modalities with CT and MRI, due to their high spatial resolution and volumetric coverage. In contrast, US is excluded due to its limited reproducibility, operator dependence, and restricted anatomical coverage.

Inclusion criteria were defined to retain studies that (i) assessed in vivo aortic deformation or motion tracking using CT or MRI; (ii) employed patient-specific approaches; and (iii) investigated motion tracking parameters.

Studies were excluded if they (i) focused on non-vascular tissues; (ii) relied solely on experimental setups or in vitro data; (iii) focused on computational or numerical modelling for deformation data; (iv) examined the effects of drugs or pharmacological treatments; or (v) focused exclusively on haemodynamics without assessing wall deformation. Although computational approaches that derived from patient-specific motion fields and subsequently applied inverse identification or numerical modelling to estimate material properties or stiffness were included, provided that the inverse methods used image-derived measurements as inputs and reported patient-specific results.

### 2.2. Study Selection and Data Extraction

Full texts of potentially eligible studies were retrieved and assessed against the inclusion criteria. Disagreements were resolved through discussion with a third reviewer (J.X.). Data extraction was performed using a standardised form capturing study characteristics (authors, year, sample size), imaging modality and parameters, segmentation methods, temporal tracking approaches, and reported biomechanical metrics.

### 2.3. Synthesis Approach

Due to substantial heterogeneity in imaging modalities, segmentation algorithms, temporal tracking methods, and the wide variety of reported biomechanical metrics across the included studies, a formal meta-analysis was not feasible. The reviewed studies employed different arterial segments, varying patient populations, and non-standardised measurement protocols, precluding meaningful statistical pooling of results. Therefore, this review adopts a narrative synthesis approach, systematically cataloguing methodological approaches and summarising findings qualitatively. Where comparable quantitative data are available and presented descriptively in tabular format to facilitate comparison, while acknowledging the limitations imposed by methodological variability.

All quantitative values were extracted as reported in the original articles. For Tables, units were harmonised, and when multiple values for the same metric were reported within a study, a simple arithmetic mean was calculated.

Given that the included studies are primarily methodological in nature, assessing imaging pipelines for arterial deformation quantification, standard quality assessment tools designed for clinical trials or diagnostic accuracy studies were not directly applicable. Instead, a custom quality assessment framework was developed based on criteria relevant to imaging-based biomechanical studies. Each study was assessed across the following four domains:

1. Sample Size Adequacy: Studies with n>19 subjects were rated as adequate, *n* = 10–19 as moderate, and n<10 as limited.
2. Time resolution: The number of time steps each article takes into account to analyse the deformation with adequate articles t>15, partial with *t* = 5–15 and inadequate with t<5.
3. Tracking Method Validation: Whether the motion tracking algorithm was validated (e.g., against phantom data, in vitro experiments, or established ground truth).
4. Reproducibility Reporting: Whether observer or automatic metrics were reported.

In addition to these four formally scored domains, we recorded descriptively whether studies reported segmentation-validation procedures, provided complete imaging-protocol details (acquisition parameters, ECG-gating, reconstruction), and included statistical reporting related to reproducibility and uncertainty; these items are summarised in the Results but were not included in the four-domain scoring table.

Each domain was scored as “Adequate” (criterion fully met), “Partial” (criterion partially met or unclear), or “Inadequate” (criterion not met). Quality assessment was performed independently by two reviewers (R.V. and B.H.), with discrepancies resolved by consensus.

Throughout the current article, we first present segmentation methods, followed by methodologies for temporal registration, and finally the metrics used to quantify deformation and their corresponding results.

## 3. Results

### 3.1. Publications Overview

**Figure 1:**
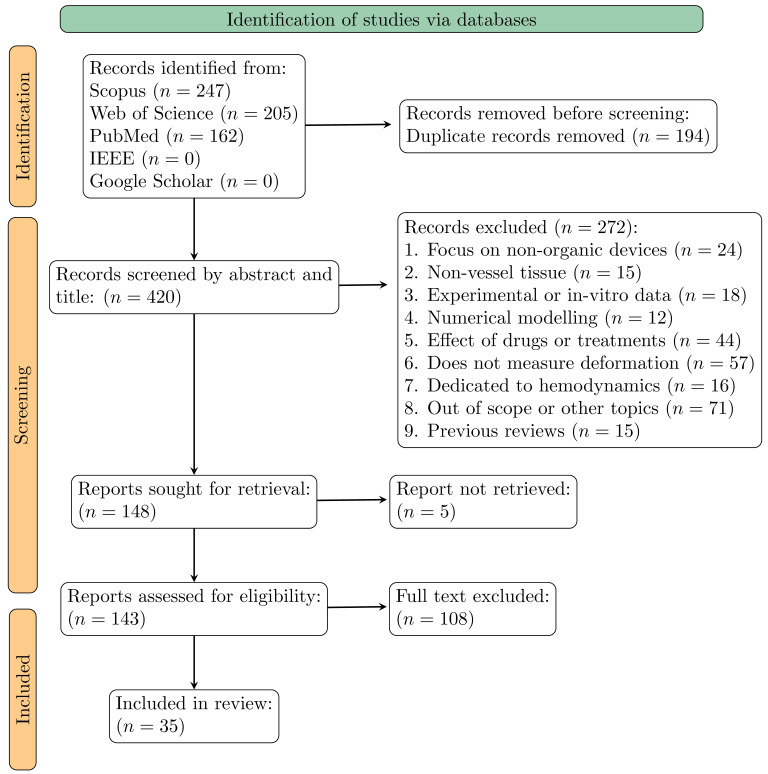
PRISMA flowchart of the systematic search strategy for the first review.

The systematic search across Scopus, Web of Science, IEEE, Google Scholar, and PubMed yielded 420 unique articles after the removal of duplicates. An initial screening of titles and abstracts led to the exclusion of 272 articles. Following a full text review of the remaining papers, a further 108 were excluded for not meeting the inclusion criteria. In the end this filtering process, detailed in Figure 1, resulted in a final cohort of 35 studies for qualitative synthesis.

**Figure 2:**
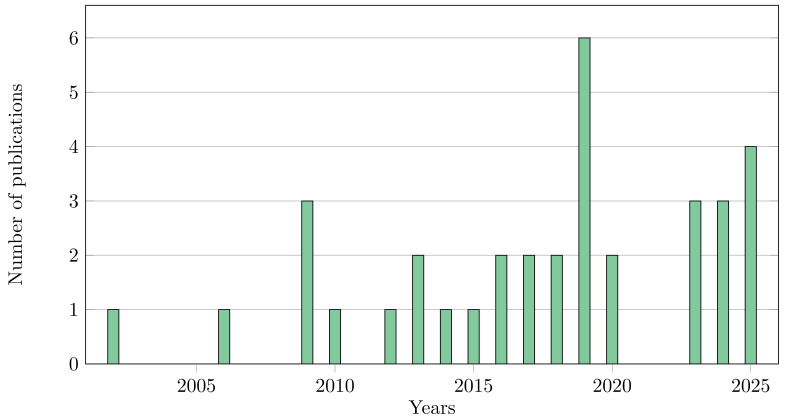
Evolution in the number of publications over recent years for the first review.

The articles selected for review were published between 2002 and 2025, with the biggest presence in 2019, as expected given the relatively recent maturation and uneven availability of the necessary imaging technologies. Figure 2 illustrates the evolution of the number of publications over time.

The quality assessment, presented in Table 1, revealed considerable methodological heterogeneity across the 35 included studies. Sample sizes ranged from 1 to 120 subjects: 11 studies (31.5%) had adequate sample sizes, 9 studies (25.7%) had moderate sample sizes, and 15 studies (42.9%) had limited sample sizes. A total of 15 studies (42.9%) reported adequate temporal resolution for the selected imaging, 7 studies (20.0%) had partial temporal resolution, and 13 studies (37.1%) had inadequate temporal resolution. Regarding validation, 15 studies (42.9%) reported segmentation validation, while 20 studies (57.1%) provided validation of their motion-tracking algorithms. Reproducibility metrics were reported in 19 studies (54.3%), and complete imaging-protocol details were provided in 24 studies (68.6%).

Of the 35 included studies, 18 (51%) utilised a form of CT, 12 (34%) used MRI, and 5 (14%) used a combination of modalities. Studies selected are more focused on large human arteries, with the cohort comprising analyses of the Abdominal Aorta Aneurysms (AAA) (nine studies), the Ascending Thoracic Aorta Aneurysm (ATAA) (5 studies), the Thoracic Aortic Aneurysm (TA) (4 studies), the femoral artery (4 studies), the carotid artery (3 studies), the Abdominal Aorta (AA) (2 studies), the Aortic Arch (AoA) (2 studies), and other major arteries in the remaining studies. This focus reflects the clinical significance and larger magnitude of motion in these major vessels.

The literature search also identified 15 relevant review articles; none overlap the scope of the current paper. Reference [15] examined in vivo stiffness measures, in particular distensibility and the β-stiffness index, and found that they are more relevant than diameter alone for detecting early aortic deformation in Marfan patients, especially when adjusted for age. Reference [16] showed that biomechanical indices, such as peak wall stress and rupture index, are elevated in symptomatic or ruptured AAA, suggesting they may indicate rupture risk diameter alone. However, it was noted that their clinical application remains limited by methodological variability and a lack of standardisation. Reference Simon [17] discussed vascular risk in hypertension, while Oluwole et al. [18] analysed the impact of mechanical forces on gene regulation. Reference Reneman et al. [19] later emphasised wall shear stress as a determinant of endothelial function and arterial remodelling. More clinically oriented contributions, including De Korte et al. [20], reviewed US-based mechanical characterisation of carotid arteries and atherosclerotic plaques, and Rong et al. [21], who discussed the value of speckle tracking echocardiography for non-invasive assessment of aortic strain. In a broader context, several reviews have examined the use of imaging and biomechanical assessment across diverse organ systems. In pulmonary research, [22] reviewed the application of CT, positron emission tomography, and MRI to investigate acute lung disease, while Wells and Liang [23] provided a comprehensive overview of US imaging. In neurology, studies have assessed the reproducibility of MRI and summarised advances in cerebrospinal fluid mechanics [24,25]. To finalise, other review articles focus on bone porosity [26], mineral bone disorders [27] and the role of connexin 43 in stem cell niches [28].

### 3.2. Image Segmentation Methods

The initial step for any pipeline for quantifying the dynamics of a vessel is a segmentation from imaging. The literature reveals a spectrum of approaches, ranging from commercial software to the development of custom and, more recently, machine learning pipelines.

Commercial and open-source software packages are prevalent. Tools such as Mimics (Materialise NV, Leuven, Belgium) [13,29,30,31], SimVascular (Open Source Medical Software Corporation, San Diego, CA, USA) [32,33,34,35], and ITK-SNAP [9,13,36,37] are frequently cited. Within these platforms, segmentation is most commonly achieved through a combination of threshold and active contour models, which remain the dominant techniques.

Several studies report the use of custom, semi-automated pipelines tailored to specific applications. A Canny edge detection algorithm pipeline was implemented in MATLAB [38]. In-house software has also been developed for specific tasks, such as direct centreline extraction from MRI [39,40], though elliptical Hough transform [41].

More recently, machine learning techniques, particularly deep learning, have been applied to vascular segmentation. Convolutional Neural Networks (CNN), with the U-Net [42,43] architecture and its variants (FC-ResNets+ residual networks), has shown promise for segmenting vessels from MRI [44]. Reference [45] performed a segmentation utilising Attention U-Net and its variants: Residual U-Net, Attention U-Net and Attention Residual U-Net, concluding that the simple U-net architecture yields better results on segmenting MRI data. Reference Du et al. [46] performed an even broader analysis across multiple U-net modalities and other relevant deep learning approaches, presenting a clear outperformance by LoGB-NET.

### 3.3. Temporal 3D Deformation Tracking Methods

Following the segmentation process, the next step is to track the deformation of the vessel over the cardiac cycle. It is important to notice the difference in imaging processes and the trade-off between the spatial and temporal resolution of the underlying imaging data. CT typically offers high spatial resolution (e.g., 0.6 mm), but its temporal resolution is often limited to around 10 phases per cardiac cycle due to constraints on radiation dose [13,31,34,37,47,48,49]. In contrast, MRI provides higher temporal resolution, often capturing up to 30 phases, but at the cost of lower spatial resolution (e.g., 1.5 mm) [45,50,51]. It is important to note that for both modalities, the time-resolved data is typically reconstructed from multiple heartbeats using Electrocardiographic (ECG)-gating to create a synthetic, representative cardiac cycle.

The majority of early studies focused on 2D tracking methods or compared 3D metrics at discrete time points (diastole and systole) rather than performing full 3D temporal tracking [34,38,39,40,41,47,48,49,52]. One of the first reports of true 3D tracking in our database was performed by [9], who applied a 3D extension of the optical flow algorithm. This voxel method assumes brightness constancy and allows for motion tracking without prior segmentation.

More advanced voxel registration algorithms have since been employed. [36] implemented a diffeomorphic Demons algorithm, a non-linear registration technique that ensures a smooth and topologically preserved transformation, enhanced with Symmetric Normalization (SyN) and Newton-Raphson iteration. Another common approach is B-spline registration, which models the displacement field using a grid of control points, offering a good balance between flexibility and smoothness. This method was used by [29], Tjahjadi et al. [43] via the open-source Elastix toolbox.

Alternatively, there are slice methods that operate on the 3D segmented geometry rather than the simple 2D. A main technique is surface parameterisation, where the vessel surface is mapped to a 2D domain for tracking. These methods generally use a cylindrical coordinate system with the centreline as the axis. This allows for consistent tracking of material points on the surface and has been used in several studies to quantify motion [13,30,31,33,53]. Another surface approach involves a non-rigid alignment of point clouds based on the Iterative Closest Point (ICP) algorithm [54]. Although these methods are computationally efficient, they are sensitive to initial alignment and noise and do not necessarily preserve the vessel’s topology. For catheter navigation, [41] employed diffeomorphic registration with spectral matching and Coherent Point Drift to align optical shape sensing data with hepatic vessel anatomy, achieving mean tip accuracy of 1.1 to 1.2 mm and shape error below 1.6 mm—performance comparable to electromagnetic tracking but compatible with MRI environments.

The reviewed literature does not contain direct comparative studies in which multiple tracking methods are evaluated on the same dataset. However, indirect comparison across studies reveals consistent methodological trade-offs. Voxel-based registration approaches generally benefit from full volumetric information and topology preservation but are sensitive to image noise and limited temporal resolution, particularly in CT. Surface-based methods offer computational efficiency and intuitive biomechanical interpretation, yet depend strongly on segmentation quality and may underestimate through-wall motion. Consequently, the suitability of a given tracking strategy is conditional on imaging modality, temporal sampling, and the biomechanical quantities of interest rather than on algorithmic class alone.

Across studies, CT pipelines favour high spatial fidelity at the expense of temporal resolution, typically reconstructing around 10 cardiac phases, whereas MRI approaches trade spatial resolution for denser temporal sampling. This trade-off has direct implications for strain estimation: coarse temporal sampling may bias peak strain values, while limited spatial resolution constrains regional heterogeneity analysis. These constraints explain part of the variability in reported metrics.

### 3.4. Metrics Used to Measure Deformation and Results

To quantify the dynamics of large vessels, a variety of metrics have been reported, which can be broadly categorised into kinematic, geometric, stiffness and material properties and other metrics.

#### 3.4.1. Kinematic Metrics

The most direct way to quantify motion is through displacement or strain. Studies relying on 2D data focus on metrics such as aortic diameter, circumferential strain, or distensibility, which are directly obtainable from cross-sectional views [38,45,48,49,50,52,55,56]. An early demonstration of wall strain measurement using cine phase-contrast MRI was reported by [57], who integrated in-plane velocity fields to compute radial displacement and subsequently derived circumferential Green-Lagrange strain. Their approach was validated in vitro with sonomicrometry, achieving displacement accuracy within 0.02 mm, and yielded in vivo thoracic and abdominal aortic strains of 0.01 to 0.08. In three dimensions, metrics based on displacement are commonly used to quantify motion over the cardiac cycle [9,31]. For instance, [58] directly tracked the x, y, z positions of defined points across time, for precise local motion. These displacement fields often serve as the basis for calculating strain. Strain metrics reveal regional aortic deformation and give more insight into wall behaviour [9,13,37,40,49]. Reference [53] also used surface expansion to compute principal stretch components for each patient.

#### 3.4.2. Geometric Metrics

Beyond deformation, several studies have quantified changes in vessel geometry. References [32,33,39,40,47], Hauguel et al. [51] reported centreline curvature and inner and outer curvatures within their Region of Interest (ROI). Reference [34] introduced the aortic arch angle using centreline normals, offering insight into arch configuration and the impact of breathing. Another study quantified femoral–popliteal vessel deformation during thigh contraction by measuring changes in aspect ratio [38].

#### 3.4.3. Stiffness and Material Properties

Although not the main focus of the present article, several identified articles in our database estimated material properties through inverse methods. References [30,31] employed the Local Extensional Stiffness Identification (LESI) methodology, which combines motion tracking with local inverse modelling to estimate patient-specific stress and strain. Other studies approached this challenge by solving an inverse pressure-area problem, where that relation derives stiffness indices like compliance and distensibility. Reference [59], developed a clever inverse method that directly utilised cine MRI pixel data to identify carotid stiffness, reporting values around 600 kPa in young, healthy subjects. Reference [60] measured the local pulse wave velocity from which they derived compliance and Young’s modulus, showing significant differences with age. Reference [61] took into account different plaque components and estimated the artery’s unloaded shape, highlighting how vital this step is for predicting stress distributions accurately. Reference [36] presented tension–extension curves for three AAA and three healthy patients, demonstrating the mechanical impact of the pathology: for a given strain, aneurysmal aortas exhibited reduced tension compared with healthy vessels. Furthermore, they proposed two novel indices, the Strain-Scaled Stiffness Index, and a hardening index that characterise the regional average stiffness and the rate of strain hardening, respectively. Their findings showed that these indices could sharply discriminate between healthy and aneurysmal arteries.

#### 3.4.4. Other Metrics

Other reported metrics include patient-specific annual growth rates, which are derived from repeated imaging and combined with clinical profiles to track disease progression over longer timescales [29,48,54]. Some studies have combined in vivo imaging with ex vivo mechanical testing and histological analysis. This allows for the validation of imaging-derived quantities such as distensibility, axial length, diameter, and volume against ground-truth measurements, providing a crucial link between non-invasive assessment and the tissue’s true mechanical behaviour [14,56].

### 3.5. Dynamic Results

Reference [14] performed mechanical testing on tissue from 33 ATAA patients and found that low distensibility (below $1\times 10^{-3}$ mmHg) was associated with increased patient age, larger aortic diameter, and adverse histological features, positioning distensibility as a sensitive marker of aneurysm progression. In the same vessel, [37] found that maximum principal strain could be as high as 30%, indicating high localised deformation. Reference [13] performed an analysis of ATAA patients and reported an age (A) decay of strain (%) as −0.06A+18.54. Furthermore, he analysed strain in patients with Tricuspid Aortic Valve (TAV) and Bicuspid Aortic Valve (BAV) and reported that while peak strain could reach 16%, it was lower in aneurysmal aortas. Younger patients and those with smaller aortic diameters exhibited higher strain, although the differences between the BAV and TAV groups were not significant. Similarly, [49] reported a drop in circumferential strain from around 10% in young individuals to 2% in the elderly, with an increase in aortic diameter. Reference [34] analysed the aortic arch and revealed that, despite significant translational movement throughout the cardiac cycle, the branching angles remain stable. This suggests a coherent motion of the arch and its branches. Surgical intervention with Thoracic Endovascular Aortic Repair (TEVAR) creates significant geometric changes, including an increase in the axial length of the ascending aorta and a decrease in arch curvature, suggesting a reduction in peak deformation post procedure [32,43,51].

Ageing profoundly impacts aortic mechanics. Reference [48] found that the ascending aortic strain decays at a rate of −0.0012A+0.1409 with age (A) and that diameter increases by about 0.12 cm per decade, systolic expansion falls from roughly 9.2% in young men to about 5.6% in older men, pressure–strain modulus increases, and strain, strain rate, and volume change all decline with age. On the Proximal Pulmonary Artery (PPA), [56] observed that the circumferential strain decay at −0.02141A+1.708 and in the axial direction as −0.00695A+0.5446, translating to an, approximately, 20% lower circumferential strain and 7% lower axial strain per decade. Notably, distensibility showed a correlation with pulmonary diffusion capacity, while axial strain correlated with right ventricular ejection fraction, linking mechanical degradation to functional decline.

In patients with AAA, dynamic metrics offer insights to complement static diameter. Reference [35] studied 22 individuals and reported a mean diameter of 46.5±8.4 mm and a mean longitudinal strain of 0.91±0.50%. Reference [36] introduced a Strain-Scaled Stiffness Index and a Hardening Index, which were strongly correlated with diameter and may better indicate disease progression. Those metrics differed by orders of magnitude between healthy and aneurysmal patients. Growth patterns in AAA are also spatially variable. Reference [54] noticed that the highest growth rates often occur near the common iliac arteries.

Studies of the peripheral vasculature reveal complex deformations, particularly during body movement. Reference [39] quantified large deformations in the Superficial Femoral Artery (SFA) during hip and knee flexion, reporting a mean axial shortening of 13% (from 22.2±3.1 to 19.2±2.5 cm) and a mean twist of $60^{\circ }$, while vessel straightness remained nearly constant at roughly 99%. Interestingly, left-right symmetry was strong for length changes but weak for twisting patterns. In a follow-up study, [40] found that these deformations were reduced in older subjects (age 50–70), with shortening dropping to about 6 to 8% by vessel segment and twist to roughly 1.3 to 2.1° per centimetre, indicating age-related stiffening. Investigating the femoral-popliteal segment during muscle contraction, [38] noted a significant change in the artery’s aspect ratio—from 0.88 ± 0.06 at rest to 0.77 ± 0.09 during contraction—and an 82% reduction in venous cross-sectional area at the distal adductor canal; paradoxically, arterial blood flow volume actually increased from 184 ± 95 to 256 ± 113 ${mL\;\min}^{-1}$ (*p* = 0.03), demonstrating how local muscle activity dynamically alters vessel geometry and haemodynamics.

On Table 2, a summarised report of multiple human vessels is presented as a function of arterial segment and age group, aggregating the main circumferential strain values reported in the reviewed studies.

## 4. Discussion

This systematic review has examined how CT and MRI are being used to quantify arterial motion and deformation. Our PRISMA analysis identified 401 articles, but only 35 studies were relevant for this topic. This demonstrates the limited development in this topic and reveals a diversity in terms of methodology. Segmentation approaches have evolved from purely manual methods to sophisticated semi-automated workflows and CNN approaches. Active contour models and threshold remain the most used vessel segmentation method, likely because they are well understood and robust. More recently, deep learning methods, particularly U-Net architectures, have shown promise for automating this step. Interestingly, [45] found that simpler U-Net architectures sometimes outperform more complex variants for MRI vessel segmentation.

Tracking vessel motion over time presents its own challenges. Early work relied on 2D methods or simple comparisons between diastole and systole. True 3D temporal tracking began to appear around 2015 with optical flow techniques. Since then, researchers have adopted a range of registration algorithms, from diffeomorphic methods that preserve topology to B-spline approaches that balance flexibility with smoothness. Surface methods offer computational efficiency but can struggle with noise. A lack of rigorous direct comparison of these different tracking strategies on the same dataset creates a limitation in result comparison.

The biomechanical patterns emerging from these studies are consistent. Ageing and aneurysmal disease stiffen the arterial wall consistently. Consequently, strain decreases, distensibility drops, and the pressure–strain relationship steepens [13,48,56]. There is also a mechanical difference in multiple regions, demonstrating tissue heterogeneity, with some regions showing much higher strain or stiffness than others [31,37]. Importantly, dynamic metrics like distensibility correlate with functional outcomes such as pulmonary diffusion capacity and ventricular function, suggesting that they capture something clinically meaningful that static diameter measurements miss. The lack of standardisation remains a significant limitation; however, systematic consideration of acquisition constraints, tracking assumptions, and validation strategies enables indirect but informative comparison across studies.

Many inverse methods for estimating wall stiffness use generic pressure waveforms rather than patient-specific measurements. Wall models often assume uniform material properties and neglect residual stresses. Reference [61] demonstrated that accounting for the unloaded vessel configuration significantly affects stress predictions, yet most studies skip this step because it is technically demanding.

This review has several limitations. First, the substantial methodological heterogeneity across studies precluded formal meta-analysis, limiting our ability to provide pooled estimates of biomechanical parameters. Second, our quality assessment revealed that many studies had limited sample sizes and lacked validation of their motion tracking algorithms, which may affect the reliability of reported metrics. Third, the diversity of arterial segments studied, ranging from the ascending aorta to peripheral vessels, makes direct comparison challenging. Fourth, publication bias cannot be excluded, as studies reporting null or negative findings may be under-represented. Finally, the rapidly evolving nature of imaging technology means that older studies may not reflect current best practices in image acquisition and processing.

Three-dimensional maps of strain and stiffness could improve rupture risk stratification for aortic aneurysms, where diameter alone has proven to be an imperfect predictor [16]. Understanding how vessels deform during the cardiac cycle could be beneficial for clinical decision making [34].

Although formal benchmarking studies are lacking, the reviewed literature allows the identification of key dimensions along which future methods should be compared: (i) spatial and temporal resolution limits imposed by acquisition; (ii) sensitivity of strain estimates to segmentation and registration error; (iii) degree of automation versus achievable accuracy; and (iv) validation strategy and reported uncertainty. Future evaluations with those topics in mind would enable meaningful comparison without requiring identical algorithms or datasets and provide a practical roadmap for community-wide benchmarking efforts.

### Standardisation Recommendations and Proposed Roadmap

The review reveals that meaningful comparison and reproducibility across studies are currently undermined by inconsistent reporting of imaging protocols, processing pipelines, and biomechanical definitions. To address this limitation, future studies quantifying in vivo arterial deformation should adhere to a minimum set of reporting requirements spanning acquisition, processing, and analysis. Imaging acquisition should be described in sufficient detail to allow replication, including modality. Subject-level information should be included as these variables directly influence arterial mechanics.

Segmentation procedures must be clearly documented, specifying the software and version used, whether methods are manual, semi-automatic, or machine-learning-based, and, for learning-based approaches, the nature of the training data. Motion-tracking or registration methodologies should be reported with equal standards, focusing on the algorithm used, reference configuration, validation/quantification metrics, and validation strategy. Biomechanical quantities should be defined mathematically rather than descriptively, explicitly stating whether strains are computed using engineering or Green-Lagrange formulations the anatomical directions considered.

Studies that incorporate loading conditions should specify whether patient-specific or generic pressure data were used, how pressure was measured or estimated, and how temporal synchronisation between pressure and image phases was achieved. Finally, uncertainty and reproducibility should be addressed explicitly through reporting of measurement error and limits.

Multicentre validation studies using harmonised acquisition and analysis protocols, together with shared benchmark datasets, are needed to assess the robustness and reproducibility of different algorithms with meaningful stratified analyses. Making reference software toolkits publicly available and organising community challenges would support transparent comparison of methods and help accelerate technical development. In parallel, targeted pilot clinical studies should examine whether deformation metrics provide added diagnostic or prognostic value for clearly defined applications, such as predicting aneurysm growth or assessing rupture risk, when compared with established clinical markers and patient follow-up. Furthermore, studies with predefined clinical endpoints and sufficiently large patient lists will be required to demonstrate clear clinical benefit beyond current standards and integration into routine clinical practice.

Wide adoption will depend not only on technical validation but also on inclusion in reporting standards and reimbursement frameworks. Throughout this process, it should be judged with a significant dataset size and diversity, reproducibility across centres, and demonstrable impact on clinical decision-making, with open sharing of data, code, and best-practice guidance as a central principle.

## 5. Conclusions

Three-dimensional imaging with CT and MRI can quantify vessel motion and deformation throughout the cardiac cycle. The methods reviewed demonstrate that dynamic biomechanical metrics such as strain, distensibility, and regional stiffness provide insights into vascular ageing and disease that go beyond what static geometry metrics do. Across multiple articles, dynamic metrics consistently show age-related stiffening, spatially heterogeneous deformation patterns, and correlations with clinical outcomes.

However, translating these research tools into clinical practice will require concerted effort. In the future, open-access benchmark datasets with ground truth measurements would accelerate method development and allow transparent comparison between described methods. Furthermore, reporting standards and nomenclature about dynamic metrics should be established. The ultimate goal is to create robust, automated workflows that can translate raw images into actionable biomechanical information at the point of care. Validation against ex vivo testing and long-term clinical follow-up will be critical for establishing the prognostic value of these metrics.

With these developments, imaging biomechanics has the potential to support patient-specific care. Regional biomechanical assessment could refine risk stratification for aneurysms, inform treatment planning for endovascular interventions, and improve our understanding of how vessels adapt to disease.

## Figures and Tables

**Table 1:** Quality assessment summary of included studies (*n* = 35).

Quality Domain	Adq.	Partial	Inadq.	Key Concerns
Sample Size Adequacy	11	9	15	Number of pilot studies
Time resolution	15	7	13	Not consistent acquisitions
Tracking Method Validation	15	-	20	Limited validation
Reproducibility reporting	19	-	16	Not automatized in some

**Table 2:** Summary of circumferential strain (mean ± standard deviation) in human arteries by age groups, as reported in the reviewed studies. Empty cells indicate that no studies in our database reported strain values for that specific combination of arterial segment and age range. The sparse coverage reflects the heterogeneity of study populations and methodological approaches in the current literature, highlighting the need for standardised reporting frameworks.

Age	30–49	50–59	60–69	70–85
Ascending Thoracic Aorta (ATA)	0.092±0.03 [48]; ≈0.108 ± 0.056 [49]	0.072 ± 0.030 [48]	0.056 ± 0.03 [48]; ≈0.030 ± 0.014 [49]	0.102 ± 0.06 [37]
AoA	≈0.082 ± 0.031 [49]; 0.08 ± 0.05 [55]		≈0.023 ± 0.016 [49]	0.061 ± 0.029 [37]
AA	0.14±0.05 [50]; 0.081 ± 0.033 [49]	≈0.065 ± 0.016 [55]	0.042 ± 0.019 [49]	0.033 ± 0.017 [37]
AAA			0.0570 ± 0.0226 [9]	0.0102 ± 0.0063 (Circ.) [35]
				0.0091 ± 0.005 (Axial) [35]

ATA—Ascending Thoracic Aorta; AoA—Aortic Arch; AA—Abdominal Aorta; AAA—Abdominal Aortic Aneurysm; Circ.—Circumferential; Axial—Axial Direction.

## Data Availability

Not available.

## Abbreviations

The following abbreviations are used in this manuscript:

AA	Abdominal Aorta
AAA	Abdominal Aorta Aneurysms
AoA	Aortic Arch
ATAA	Ascending Thoracic Aorta Aneurysm
BAV	Bicuspid Aortic Valve
CNN	Convolutional Neural Networks
CT	Computed Tomography
CTA	Computed Tomography Angiography
CVD	Cardiovascular Diseases
DTA	Descending Thoracic Aorta
ECG	Electrocardiographic
ICP	Iterative Closest Point
LDDMM	Large Deformation Diffeomorphic Metric Mapping
LESI	Local Extensional Stiffness Identification
MRI	Magnetic Resonance Imaging
OSF	Open Science Framework
PPA	Proximal Pulmonary Artery
PRISMA	Preferred Reporting Items for Systematic Reviews and Meta-Analyses
ROI	Region of Interest
SFA	superficial Femoral Artery
SyN	Symmetric Normalisation
TA	Thoracic Aortic Aneurysm
TAV	Tricuspid Aortic Valve
TEVAR	Thoracic Endovascular Aortic Repair
US	Ultrasound
